# Study protocol: analysis of gene expression profiles in peripheral blood and tumor tissue of Colombian patients diagnosed with epithelial ovarian cancer

**DOI:** 10.3389/fonc.2026.1839127

**Published:** 2026-05-22

**Authors:** Elizabeth Vargas-Castellanos, William Torres, Julian C. Riano-Moreno

**Affiliations:** 1 Hospital Universitario Mayor – Méderi, Universidad del Rosario, Bogotá D.C., Colombia; 2 Department of Pathology, Instituto Nacional de Cancerología, Bogotá D.C., Colombia; 3 Faculty of Medicine, Universidad Cooperativa de Colombia, Villavicencio, Colombia; 4 Department of Bioethics, Universidad el Bosque, Bogotá D.C., Colombia

**Keywords:** gene expression, genetics, germline variants, ovarian cancer, transcriptome

## Abstract

**Background:**

Molecular characterization of epithelial ovarian cancer has been conducted predominantly in populations of European ancestry, leaving Latin American populations significantly underrepresented. Colombian patients, characterized by a unique admixture of Amerindian, European, and African ancestries, lack integrated genomic and transcriptomic studies. This study aims to characterize germline variants and gene expression profiles in peripheral blood and tumor tissue from Colombian patients with epithelial ovarian cancer, to identify clinically relevant molecular alterations.

**Methods:**

This is a single-center, prospective, cross-sectional analytical study that will include 30 Colombian women diagnosed with epithelial ovarian cancer (stages I–III) and 30 healthy controls, recruited at Hospital Universitario Mayor Méderi in Bogotá. The study will be conducted in three phases (1): standardized collection of paired tumor tissue and peripheral blood samples (2); germline genetic profiling using a 106-gene hereditary cancer next-generation sequencing panel, with variant classification according to international guidelines; and (3) transcriptomic analysis through RNA sequencing in both tissue and blood samples. Differential gene expressions, functional enrichment analysis, tumor microenvironment characterization, and integrated germline-transcriptomic analyses will be performed. Ethical approval was obtained from the Ethics Committee of Universidad del Rosario, and written informed consent will be obtained from all participants before enrollment.

**Discussion:**

This study will generate the first integrated germline and transcriptomic dataset for epithelial ovarian cancer in a Colombian population. The identification of clinically relevant genetic variants and expression patterns may contribute to improved molecular stratification and support the development of precision oncology approaches. Additionally, this work aims to provide foundational evidence for the implementation of population-specific biomarkers and to reduce existing disparities in cancer genomic research in Latin America.

## Introduction

Ovarian cancer (OC) is one of the leading causes of cancer-related mortality among women worldwide, with approximately 313,959 new cases and 207,252 deaths reported globally in 2022 (1). The 5-year relative survival rate is approximately 45%, largely reflecting the persistent challenge of late-stage diagnosis, as more than 70% of cases are detected at FIGO stages III–IV when curative options remain limited (2). In Colombia, the age-standardized incidence rate (ASR) of 6.7 per 100,000 women and a mortality ASR of 4.1 per 100,000 correspond to 2,253 new cases and approximately 1,445 deaths in 2022—figures that underscore the relevance of OC as a public health priority in a setting where population-based screening is absent (1). The current diagnostic standard relies on the CA125 serum glycoprotein, which lacks sufficient specificity due to its elevation in benign conditions such as endometriosis, pelvic inflammatory disease, and liver disease (3). Standard first-line treatment consists of cytoreductive surgery combined with platinum-based chemotherapy (4), and the emergence of poly(ADP-ribose) polymerase (PARP) inhibitors has improved outcomes in patients harboring pathogenic germline variants in BRCA1/2 (4), underscoring the growing clinical relevance of integrating germline profiling into the molecular characterization of EOC.

Epithelial ovarian cancer (EOC) is a heterogeneous malignancy comprising five principal histotypes according to the 2020 WHO Classification of Female Genital Tumours: high-grade serous carcinoma (HGSC, 70–80%), endometrioid carcinoma (10%), clear cell carcinoma (10%), mucinous carcinoma (3%), and low-grade serous carcinoma (<5%), each constituting a distinct disease entity with a unique molecular profile and treatment response (5–7). Gene expression profiling has been instrumental in the molecular characterization of EOC: seminal work by Tothill et al. (8) and the comprehensive genomic analyses of The Cancer Genome Atlas (TCGA) (9) established foundational transcriptomic classifications with prognostic significance. However, both datasets were predominantly derived from North American and European populations. Latin American patients represent fewer than 0.38% of participants in genomic studies to date (12), and their cancer genomic data remain profoundly scarce—a gap particularly consequential given that Colombian and broader Latin American populations exhibit a unique genetic admixture of Native American, European, and African ancestries that may harbor population-specific molecular patterns not captured by existing biomarker frameworks (10–12). Recent Colombian studies have begun to document the local hereditary cancer variant landscape (13, 14), confirming both its clinical relevance and its divergence from European reference databases. Emerging evidence from other tumor types confirms that Latin American genetic ancestry is associated with distinct somatic and germline mutational profiles (11, 13), suggesting that direct extrapolation of Eurocentric biomarker candidates to this population may be inadequate.

Transcriptomic profiling through RNA sequencing (RNA-seq) has emerged as a powerful strategy to identify dysregulated signaling pathways and uncover molecular subtypes in EOC (15, 16). While most studies have focused exclusively on tumor tissue, analysis of the peripheral blood transcriptome has gained traction as a complementary minimally invasive approach, given that alterations in circulating immune cells and cell-free RNA reflect systemic tumor–host interactions and may serve as accessible surrogates for tumor biology (17–19). This approach aligns with the liquid biopsy paradigm, which enables non-invasive molecular profiling of tumor-derived components in blood for diagnostic, prognostic, and monitoring purposes (17). Importantly, a substantial proportion of existing molecular studies in OC have been conducted within the framework of hereditary breast-ovarian cancer syndrome (HBOCS), focusing on patients with pathogenic germline variants in *BRCA1/2* or *PALB2* (4, 14, 20). The present study, by contrast, is centered exclusively on EOC patients regardless of hereditary background; the germline component is specifically designed to determine whether the transcriptomic phenotype observed is associated with an identifiable germline predisposition or whether it constitutes an independent molecular signature of EOC in this population.

This study aims to describe and compare the transcriptomic profiles of tumor tissue and peripheral blood in Colombian patients diagnosed with EOC, and to evaluate their association with clinical and histopathological features. By integrating germline profiling with dual-compartment transcriptomic analysis in a population not previously characterized at this molecular level, this work seeks to identify potential population-specific molecular patterns that may inform biomarker discovery and precision oncology strategies for EOC in Colombia. It is recognized that tumor tissue and peripheral blood constitute distinct cellular environments, precluding assumptions of transcriptomic equivalence between these compartments. Rather, the presence of specific signatures in the transcriptome of circulating blood is considered complementary—but not necessarily identical—to signaling pathways active within the tumor microenvironment. This comparative approach seeks to assess whether certain dysregulated pathways, such as those related to inflammation or immune evasion, demonstrate a functional correlation enabling the use of blood as a surrogate for tumor–host interactions.

To our knowledge, this represents the first integrated germline and transcriptomic study of EOC conducted in a Colombian population, contributing evidence to address the critical underrepresentation of Latin American patients in global cancer genomic research.

## Methods and analysis

### Study design

This is a single-center, observational, prospective, cross-sectional analytical study with group comparison, conducted at Hospital Universitario Mayor Méderi (Bogotá, Colombia). Paired tumor tissue and peripheral blood samples will be collected from patients newly diagnosed with EOC and from healthy women, enabling comparative transcriptomic profiling between compartments and between groups.

### Sample size

Based on institutional data from the 2022 oncology cohort at Hospital Universitario Mayor Méderi — where 58 OC patients were identified, of whom 80% had epithelial tumors and 65% presented in FIGO stages I–III — a target of 30 patients per group is estimated for the current study period. This sample size is consistent with comparable published exploratory transcriptomic studies (18, 19) and provides approximately 80% power to detect differentially expressed genes with a fold-change ≥ 2 and FDR < 0.05 under assumptions appropriate for RNA-seq designs (21). Given the absence of prior population-specific variance estimates for Colombian EOC patients, this study is designed as a necessary first step to generate the foundational data required for a fully powered future investigation.

### Study population

The study population comprises two groups. Group A consists of 30 adult Colombian women with a confirmed histopathological diagnosis of EOC in FIGO stages I, II, or III, who are under the care of the Gynecologic Oncology Service at Hospital Universitario Mayor Méderi (Bogotá, Colombia). Stage IV disease was excluded given that patients at this stage typically require neoadjuvant chemotherapy prior to surgery, which would preclude collection of treatment-naïve samples and introduce confounding effects on both germline and transcriptomic analyses. Group B consists of 30 healthy Colombian women without a personal history of cancer or active comorbidities, recruited through an internal call directed at employees, collaborators, and trainees of the Méderi Hospital Network, and through referrals by Méderi staff. Inclusion and exclusion criteria for both groups are detailed in **Table 1**.

**Table 1:** Inclusion and exclusion criteria for Groups A and B.

Criteria	Group A — EOC patients	Group B — healthy women
Inclusion	Native Colombian women*	Native Colombian women*
	Adult women (≥18 years)	Adult women (≥18 years)
	Confirmed EOC diagnosis by pathology report (FIGO stages I–III)	No personal history of or concurrent cancer
	Availability of paired peripheral blood and fresh/frozen tumor tissue	No active comorbidities
	Treatment-naive at time of sample collection (no prior chemotherapy, immunotherapy, radiotherapy, or targeted therapy)	Voluntary participation with signed informed consent
	Under care of Gynecologic Oncology Service at study site	Active infectious disease at time of sample collection
Exclusion	Concurrent or prior other malignancy	
	Pregnancy	Autoimmune disease or use of immunomodulatory drugs (corticosteroids, biologics)
	Documented neuropsychiatric disorder	Pregnancy
		Recent surgery (within 4 weeks prior to collection)
		Documented neuropsychiatric disorder

*Individuals born in Colombia, with both parents born in Colombia, as a proxy for Andean Colombian genetic ancestry.

### Sampling method

A non-probabilistic consecutive convenience sampling strategy will be employed, selecting all eligible patients meeting the predefined criteria during the recruitment period at the study site. Although this approach may have limitations in external representativeness, it is appropriate for exploratory molecular profiling studies in populations where no prior data exists and is consistent with analogous published studies in this field (18, 19).

### Data collection and monitoring

Clinical and demographic data will be collected by a designated research assistant through review of medical records and entered the REDCap platform (22, 23), which ensures secure, structured data storage and audit trail documentation throughout the recruitment period. Variables collected are summarized in **Table 2**.

**Table 2:** Demographic, clinical, and histopathological variables collected per participant.

Domain	Variable
Demographics	Age, year of birth; birth and residence department; socioeconomic stratum
Clinical	Stage (FIGO classification); date of diagnosis; symptoms at presentation; menopausal status and age at menopause; hormone replacement therapy (type and duration); obstetric history (number of pregnancies, deliveries, age at first delivery); contraceptive method and duration; family history of cancer; associated autoimmune, chronic, and systemic diseases; serum tumor markers (CA125, CA19-9, CEA); current medications; surgical date; adjuvant treatment and chemotherapy regimen; vital status and date of death
Pathological	EOC histotype (pathology report); degree of tumor differentiation (grade); immunohistochemistry panel: ER, PR, HER2, Ki67, PAX8, p16, WT1, p53, BRCA1, BRCA2 (when available)

CA125, Cancer Antigen 125; CA19-9, Cancer Antigen 19-9; CEA, Carcinoembryonic Antigen; ER, Estrogen Receptor; PR, Progesterone Receptor; HER2, Human Epidermal Growth Factor Receptor 2; Ki67, Marker of Proliferation Ki-67; PAX8, Paired Box Gene 8; p16, Cyclin-Dependent Kinase Inhibitor 2A; WT1, Wilms Tumor 1; p53, Tumor Protein p53.

**Figure 1:**
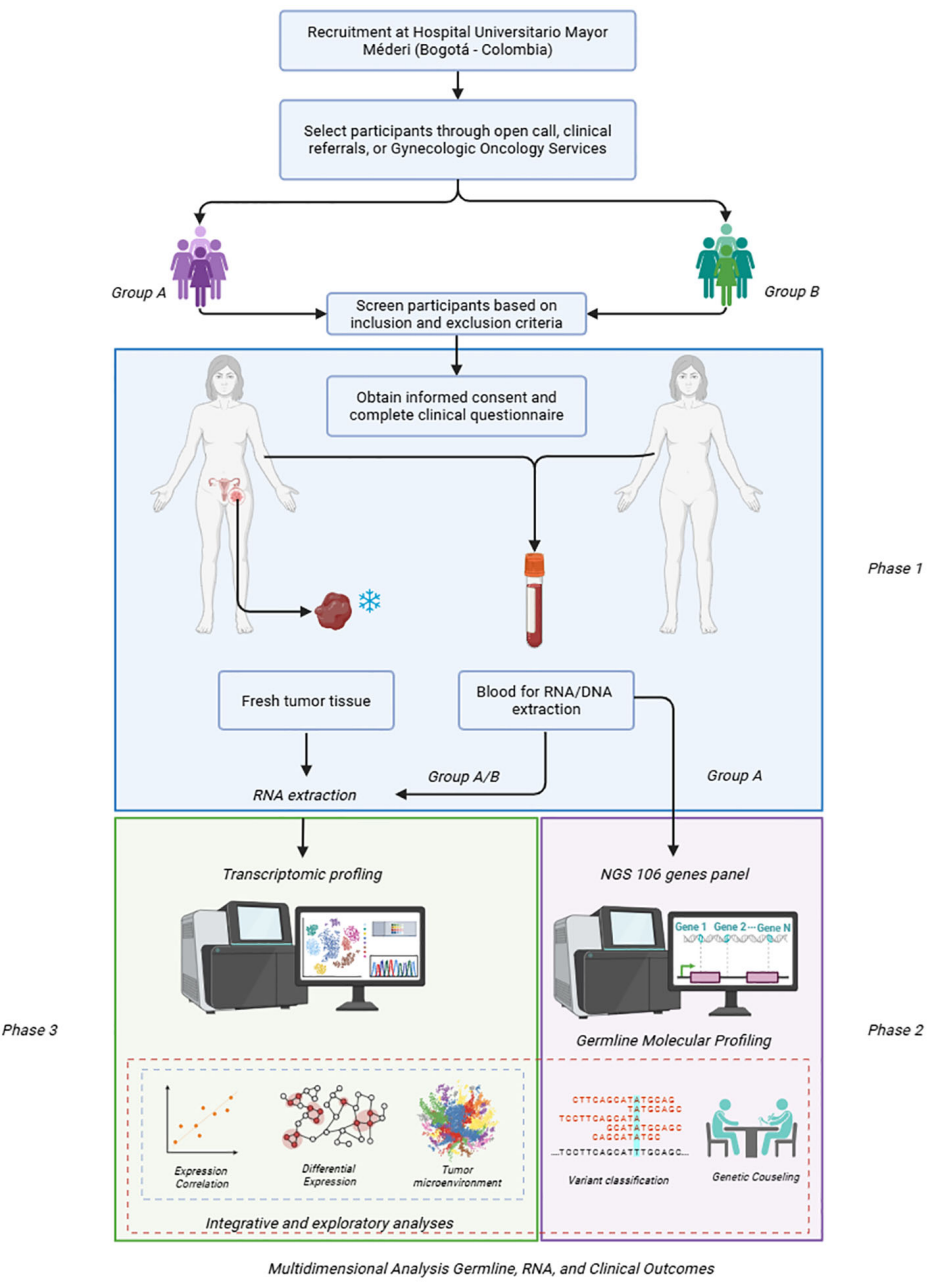
Study design and molecular analysis workflow. Created in https://BioRender.com.

The methodological process for the project will be developed as follows (**Figure 1**).

### Phase 1: biological sample collection

#### Group A

Sample collection will be performed on the day of surgery in patients with high clinical suspicion of malignancy, defined as the presence of adnexal masses classified as high-risk by the IOTA ADNEX model or a Risk of Malignancy Index (RMI = U × M × CA125) ≥ 200 (24). Prior to any oncologic treatment, three 5 mL peripheral blood samples will be collected in EDTA tubes. Tumor tissue will be obtained intraoperatively; a minimum volume of 0.5 cm³ (approximately 40 mg) is required to ensure adequate RNA yield for transcriptomic analysis. Only patients with confirmed histopathological diagnosis of EOC will proceed to molecular analyses; samples from patients with a negative pathological result will be discarded in accordance with institutional biosafety protocols.

#### Group B

Blood collection will be performed by scheduled appointment at the research module of the clinical laboratory at Hospital Universitario Mayor Méderi, with transportation costs covered by the research budget.

##### Sample processing and storage

All samples will be processed and stored in the clinical laboratory research area following institutional standard operating procedures.

- *Fresh tumor tissue*: specimens will be immediately rinsed with RNase-free water, placed in 15 mL tubes containing RNAlater solution (0.5 cm³ tissue per 5 mL RNAlater), and stored at −80 °C until processing.
- Blood for RNA extraction: samples will be processed using TRIzol reagent (TRIzol:blood ratio 3:1 v/v) in 15 mL tubes, vigorously homogenized for 1–2 minutes, incubated at room temperature for 5 minutes, aliquoted into 2 mL cryovials, and stored at −80 °C. Since whole blood contains high proportions of globin mRNA, which may represent a dominant fraction of the peripheral blood transcriptome, downstream bioinformatic analyses will include computational removal of globin transcripts prior to differential expression analysis, using in silico filtering of ENSG00000206172 (HBA1), ENSG00000188536 (HBA2), ENSG00000244734 (HBB) and related loci. This approach has been validated as a reliable alternative to wet-lab globin depletion in studies using TRIzol-preserved whole blood (Baumann et al., BMC Genomics, 2019).
- Blood for DNA extraction: EDTA tubes for germline analysis will be stored at 4 °C until processing.

##### Sample shipment

Packaging will be performed by an IATA-certified bacteriologist complying with international regulations for biological materials. Samples will be shipped on dry ice through a certified carrier under supervision of the Principal Investigator.

### Phase 2: germline molecular profiling (group A)

Germline molecular profiling will be performed in all Group A patients using a validated,comprehensive next-generation sequencing (NGS) panel of 106 genes commonly associated with hereditary cancer syndromes linked to ovarian cancer risk (**Supplementary Table 1**). The panel encompasses multiple DNA repair and cancer susceptibility pathways, including HRR (homologous recombination repair) (*BRCA1*, *BRCA2*, *PALB2*, *BRIP1*, *RAD51C*, *RAD51D*, *ATM*, *CHEK2*, and related genes), Fanconi anemia pathway genes (*FANCA–FANCM*), mismatch repair (MMR) and Lynch syndrome genes (*MLH1*, *MSH2*, *MSH6*, *PMS2*, *MSH3*, *MLH3*, *PMS1*, *EPCAM*) among others. Sequencing will be performed on Illumina platforms at a certified external sequencing facility (Sacramento, CA, United States) with a minimum mean coverage of 100× for germline variant calling.

Variant classification will follow the American College of Medical Genetics and Genomics (ACMG) and the Association for Molecular Pathology (AMP) framework. A systematic manual curation of all VUS will be conducted in alignment with ACMG guidelines and recommendations. This process will employ publicly available variant assessment tools, ensuring that their default criteria are modified and interpreted in accordance with the most recent ClinGen guidance for variant- and gene-specific evaluation. Additional criteria—such as patient phenotype correlation, familial segregation, or other pertinent—will be applied when appropriate to enhance variant interpretation. The classification of VUS will also incorporate the cold and hot variant framework established by CanVIG-UK (Cancer Variant Interpretation Group UK), facilitating more accurate categorization within the context of cancer-related variants.

Patients with confirmed pathogenic or likely pathogenic variants will receive pre- and post-test genetic counseling and will be referred for clinical follow-up to the institution’s Clinical Genetics Service, with implementation of a surveillance and management protocol in accordance with the identified hereditary syndrome; coordination of extended follow-up will involve the participant’s healthcare insurer as applicable. The management of incidental findings unrelated to the primary study indication will follow institutional policies and will be disclosed to participants in accordance with the pre-test informed consent process.

### Phase 3: transcriptomic profiling

Total RNA will be extracted from stored tumor tissue (Group A) and peripheral blood samples (Groups A and B) using standardized protocols. RNA quality will be assessed by spectrophotometry (Nanodrop; A260/280 ratio ≥ 1.8) and capillary electrophoresis (Bioanalyzer or TapeStation). Only samples meeting minimum quality thresholds — RNA Integrity Number (RIN) ≥ 7 for tumor tissue and RIN ≥ 6 for peripheral blood, and DV200 value > 66:1% with a minimum concentration of 50 ng/μL — will proceed to library preparation.

Strand-specific RNA-seq libraries will be constructed following standard protocols. Sequencing will be performed on Illumina platforms at a certified external sequencing facility (Sacramento, CA, United States) targeting a minimum of 30 million paired-end reads per sample (150 bp read length), consistent with recommended depth for differential gene expression analyses. A sequencing depth of 30 million reads was determined through statistical power simulations to ensure reliable detection of fold-changes exceeding two in genes with moderate expression levels. While increased depth is preferable for isoform quantification, this limitation will be addressed by implementing the Salmon algorithm. Salmon utilizes a probabilistic inference model that accounts for fragment bias, thereby enhancing the precision of read assignment to specific isoforms, even at conventional sequencing depths.

### Current study status

Recruitment for this single-center, prospective observational study is currently ongoing at the Gynecologic Oncology Service of the Hospital Universitario Mayor Méderi (Bogotá, Colombia). Ethical approval was obtained from the institutional research ethics committee prior to study initiation. To date, participant identification and eligibility screening are being conducted among women with a new histopathological diagnosis of EOC (FIGO stages I–III) who are candidates for primary surgical management. Collection of paired peripheral blood and tumor tissue samples from treatment-naïve patients is actively underway.

In parallel, recruitment of healthy control participants without a personal history of cancer or active comorbidities is being performed through an internal institutional call directed to employees, collaborators, and trainees of the Méderi Hospital Network. Clinical and demographic data are being systematically recorded using the REDCap electronic data capture platform to ensure secure storage and audit trail documentation. To minimize potential selection bias and confounding effects, controls will be frequency-matched to cases based on age. This matching strategy is intended to reduce the impact of age-related differences in gene expression, particularly in peripheral blood transcriptomic analyses.

Biological sample processing and storage are being conducted in accordance with institutional standard operating procedures. Arrangements for international shipment of biological material for external sequencing analyses are currently in progress in compliance with national regulatory requirements. Participant recruitment is expected to continue until the predefined target sample size for both study groups is achieved.

### Patient and public involvement

Patients and the public were not involved in the design, conduct, reporting, or dissemination plans of this research. This study involves the analysis of clinical data and biological samples obtained as part of routine clinical care. Therefore, no direct patient or public involvement was included in the development of the study protocol. Results from this research will be disseminated through scientific publications and presentations.

### Statistical and bioinformatic analysis

#### Clinical data

Statistical analysis will be performed in R (v4.x). Continuous variables will be assessed for normality using the Shapiro–Wilk test and reported as mean ± standard deviation or median (IQR). Categorical variables will be reported as frequencies and percentages. Between-group comparisons will use Student’s t-test or Mann–Whitney U test for continuous variables, and chi-square or Fisher’s exact test for categorical variables. A two-sided p-value < 0.05 will be considered statistically significant.

#### Bioinformatic pipeline

Raw FASTQ files will undergo quality control assessment using FastQC (v0.11.x) and MultiQC (v1.x), with adapter trimming performed using Trimmomatic (v0.39). Trimmed reads will be aligned to the human reference genome (GRCh38/hg38) using STAR (v2.6.1d). Gene-level read counts will be generated using featureCounts (v1.6.2) with GENCODE annotation (release 29). In parallel, transcript-level quantification will be performed using Salmon (v0.11.3) based on GENCODE annotation (release 28), enabling isoform-level expression estimates. The parallel approach enables one process to verify genomic alignment integrity and maintain quality control, while a separate process delivers precise isoform quantification through probabilistic modeling. Combining these methodologies allows for effective discrepancy resolution, emphasizes abundance normalized by transcript length, and consequently refines the detection of authentic biological signals over technical artifacts.

Differential gene expression analysis will be conducted using DESeq2 (v1.x), comparing EOC patients versus healthy controls (peripheral blood) and tumor tissue versus peripheral blood (within Group A). Genes with an absolute log_2_ fold-change > 1 and adjusted p-value (Benjamini–Hochberg FDR) < 0.05 will be considered differentially expressed. Functional enrichment analyses will be performed using Gene Ontology (GO) biological process, KEGG, and Reactome pathway databases via the clusterProfiler R package. Variant calling from RNA-seq data (SNPs and indels) will be performed using GATK best-practices pipelines. All raw sequencing data will be deposited in the NCBI Gene Expression Omnibus (GEO) repository.

#### Integrative and exploratory analyses

Prior to differential expression analysis, potential batch effects attributable to sample processing date, sequencing run, or RNA quality will be assessed and corrected using the ComBat-seq algorithm (sva R package). Principal component analysis (PCA) will be performed on normalized expression matrices to evaluate sample clustering, detect outliers, and assess the contribution of technical and biological covariates. Integrative analyses will be conducted to examine associations between transcriptomic profiles, germline variant status, and clinicopathological variables (FIGO stage, histotype, tumor grade, and serum CA125), using multivariate regression models and unsupervised hierarchical clustering. Correlation between gene expression patterns and germline findings will be explored to evaluate whether the transcriptomic phenotype is associated with a specific hereditary predisposition or constitutes an independent molecular signature. Tumor microenvironment (TME) composition will be estimated from RNA-seq data using cell-type deconvolution algorithms (CIBERSORT and/or TIMER), enabling inference of immune cell infiltration patterns and their association with germline status and clinical variables.

## Discussion

This protocol addresses a critical gap in the molecular oncology literature: the near-total absence of integrated germline and transcriptomic data on EOC in Latin American populations. Despite the substantial contribution of large consortia such as TCGA and the Ovarian Cancer Cohort Consortium to the molecular characterization of this disease, their reference datasets are derived overwhelmingly from patients of European ancestry (1, 2). Latin American populations exhibit a unique and complex admixture of Amerindian, European, and African ancestral components, with distinct germline variant spectra and gene expression regulation patterns that cannot be reliably inferred from Eurocentric references (3, 4). Recent Colombian studies have begun to document the local hereditary cancer variant landscape (13, 14), confirming both its clinical relevance and its divergence from European reference databases — providing the contextual foundation on which the present protocol builds by extending the scope to EOC-specific integrated transcriptomic profiling.

Three features distinguish this protocol from prior studies in the field. First, germline testing is applied systematically to all enrolled patients using a comprehensive 106-gene panel — in contrast to most published studies, which either restrict testing to *BRCA1/2*, or are not exclusive to ovarian cancer and include only marginal proportions of EOC patients (10). This unselected, broad-panel design enables unbiased population-level inference on hereditary variant frequency and spectrum in Colombian EOC patients, data that are urgently needed to support evidence-based counseling and cascade testing programs in this setting. Second, the simultaneous profiling of tumor tissue and peripheral blood transcriptomes enables the characterization of both intrinsic tumor biology and systemic immune and inflammatory responses associated with malignancy — a dual-compartment approach with recognized biomarker potential (5–7) that has not been applied to this population. A recent Colombian study demonstrated the yield of integrated transcriptomic and microenvironmental profiling in hereditary versus sporadic triple-negative breast cancer (25), establishing a regional methodological precedent that the present protocol extends to EOC. Third, the integration of germline variant status with transcriptomic profiles from both compartments, linked to comprehensive clinicopathological variables, allows for the exploration of whether hereditary predisposition is associated with a distinct molecular phenotype. Complementary sub-analyses will include tumor microenvironment deconvolution (CIBERSORT/TIMER) to infer immune cell infiltration patterns and their association with germline status — an analysis that has not been previously conducted in Colombian EOC patients.

The translational implications are concrete and extend across multiple horizons. In the immediate term, systematic germline testing will generate actionable clinical results for participants and their families; identification of pathogenic variants in HRR genes — particularly *BRCA1*, *BRCA2*, and *PALB2* — will directly inform eligibility for PARP inhibitor therapy, an indication whose implementation in Colombia remains constrained by the absence of local prevalence data to guide testing policies. The integration of germline status with transcriptomic profiles may further reveal whether HRR-deficient patients in this population harbor a distinct gene expression signature that could refine therapeutic stratification beyond germline testing alone. In the medium term, blood-based transcriptomic signatures may support the development of minimally invasive monitoring tools adapted to the access constraints of the Colombian healthcare system. The peripheral blood transcriptomic data generated by this study serves as a foundational discovery layer to identify potential candidates for future liquid biopsy frameworks. While peripheral blood gene expression is known for its high biological variability, the differentially expressed genes and pathways identified here may provide preliminary candidates for subsequent, more targeted validation using proteomic or cell-free RNA approaches. Building upon these initial observations, future longitudinal studies in independent Colombian cohorts will be essential to integrate multi-omic platforms—such as circulating tumor DNA (ctDNA) and microRNA profiling—to refine and confirm the clinical utility of these signatures. Ultimately, while acknowledging the inherent constraints of this first-generation pilot, the establishment of locally derived molecular reference data represents a critical step toward adapting precision oncology frameworks—historically based on Eurocentric evidence—to the specific biological and genetic context of the Colombian and Andean populations.

Potential limitations include the exploratory sample size, which limits power for subgroup analyses and detection of low-frequency variants; the cross-sectional design, which precludes assessment of longitudinal molecular dynamics; and the single-center setting, which may restrict external generalizability. Findings will require validation in independent Colombian and Latin American cohorts. Nevertheless, these constraints are inherent to a necessary first-generation study, and the protocol is designed to generate the variance estimates and preliminary effect sizes required for a fully powered future investigation.

### Patient and public involvement

No patients or members of the public were involved in the design or planning of this protocol, given its exploratory and highly technical nature. Accessible lay summaries of study findings will be made available to participants upon study completion, and patient involvement will be prioritized in the design of any future interventional studies arising from these results.
