# Supplementary material for: Study protocol: analysis of gene expression profiles in peripheral blood and tumor tissue of Colombian patients diagnosed with epithelial ovarian cancer
Source: Front Oncol. 2026 May 22;16:1839127. doi: 10.3389/fonc.2026.1839127 (PMC13236929; doi:10.3389/fonc.2026.1839127)
Supplement: Supplementary file 1 [file Table1.docx]

**Supplementary Table S1. Comprehensive hereditary cancer gene panel used for germline molecular profiling**

| Pathway / Hereditary Syndrome | Genes |
| --- | --- |
| Homologous Recombination Repair (HRR) | *ATM, BARD1, BRCA1, BRCA2, BRIP1, CHEK2, MRE11, NBN, PALB2, RAD50, RAD51C, RAD51D, SLX4* |
| Fanconi Anemia | *FANCA, FANCB, FANCC, FANCD2, FANCE, FANCF, FANCG, FANCI, FANCL, FANCM* |
| Mismatch Repair / Lynch Syndrome | *EPCAM, MLH1, MLH3, MSH2, MSH3, MSH6, MUTYH, PMS1, PMS2* |
| Nucleotide Excision Repair | *DDB2, ERCC2, ERCC3, ERCC4, ERCC5, XPA, XPC* |
| Cell Cycle and Tumor Suppression | *CDK4, CDKN1B, CDKN2A, PTEN, RB1, TP53* |
| Chromosomal Instability / Spindle Assembly | *BLM, BUB1, BUB1B, BUB3* |
| Gastrointestinal Polyposis Syndromes | *APC, AXIN2, BMPR1A, CDH1, SMAD4, STK11* |
| Renal and Metabolic Tumor Syndromes | *BAP1, FH, FLCN, MET, SETD2, TSC1, TSC2, VHL* |
| Hereditary Endocrine and Neuroendocrine Tumors | *AIP, MAX, MEN1, RET, SDHA, SDHAF2, SDHB, SDHC, SDHD, TMEM127* |
| Hereditary Melanoma and Skin Tumors | *MITF, NF1, NF2, PTCH1, SUFU* |
| Hematological Malignancy Predisposition | *CEBPA, GATA2, RUNX1* |
| Neural Crest and Neuroendocrine Tumors | *ALK, KIF1B, NTRK1, PDGFRA, PHOX2B* |
| Other Hereditary Cancer Predisposition Syndromes | *CDC73, CYLD, DICER1, DIS3L2, EGFR, ELAC2, EXT1, EXT2, HNF1A, HOXB13, KIT, NSD1, PALLD, PRKAR1A, PRSS1, RHBDF2, RNASEL, SMARCB1, SRD5A2, WT1* |

*HRR: Homologous Recombination Repair*
